# Supplementary material for: Factors Associated with Reported COVID-like Symptoms and Seroprevalence Data Matched with COVID-like Symptoms in Slums and Non-Slums of Two Major Cities in Bangladesh
Source: Healthcare (Basel). 2023 May 16;11(10):1444. doi: 10.3390/healthcare11101444 (PMC10218417; doi:10.3390/healthcare11101444)
Supplement: Supplementary file 1 [file healthcare-11-01444-s001.zip › healthcare-2345083-supplementary.pdf]

healthcare

MDPI

**Supplementary table file**

**Supplementary Table S1.** Bivariate logistic regression estimates of reported COVID-like symptoms by selected socio-demographic covariates.

| Factors | Reported COVID-like Symptoms | | | | | |
|---|---|---|---|---|---|---|
| | Combined | | Slum Area | | Non-Slum Area | |
| | OR (95% CI) | *p*-Value | OR (95% CI) | *p*-Value | OR (95% CI) | *p*-Value |
| **City** | | | | | | |
| Dhaka | 1 | | 1 | | 1 | |
| Chattogram | 1.21 (1.12, 1.32) | <0.001 | 1.54 (1.37, 1.73) | <0.001 | 0.88 (0.78, 0.99) | 0.035 |
| **Area** | | | | | | |
| Slum | 1 | | - | | - | |
| Non-slum area | 1.30 (1.19, 1.41) | <0.001 | - | | - | |
| **Age group (years)** | | | | | | |
| 10-17 | 1 | | 1 | | 1 | |
| 18-59 | 1.31 (1.17, 1.47) | <0.001 | 1.34 (1.15, 1.56) | <0.001 | 1.27 (1.07, 1.50) | 0.005 |
| 60 or more | 1.33 (1.11, 1.60) | 0.002 | 1.32 (1.01, 1.72) | 0.042 | 1.26 (0.98, 1.62) | 0.069 |
| **Sex** | | | | | | |
| Female | 1 | | 1 | | 1 | |
| Male | 1.06 (0.98, 1.16) | 0.165 | 0.92 (0.82, 1.03) | 0.165 | 1.31 (1.16, 1.49) | <0.001 |
| **Education (years of schooling)** | | | | | | |
| None | 1 | | 1 | | 1 | |
| 1-4 | 1.21 (1.04, 1.40) | 0.011 | 1.21 (1.03, 1.41) | 0.019 | 1.26 (0.83, 1.91) | 0.273 |
| 5-9 | 1.32 (1.17, 1.49) | <0.001 | 1.28 (1.11, 1.47) | 0.001 | 1.46 (1.04, 2.07) | 0.033 |
| 10 or more | 1.70 (1.50, 1.92) | <0.001 | 1.43 (1.14, 1.79) | 0.002 | 1.87 (1.33, 2.62) | 0.000 |
| **Occupation** | | | | | | |
| Not-working/unem-ployed | 1 | | 1 | | 1 | |
| Service | 1.67 (1.40, 1.99) | <0.001 | 1.70 (1.36, 2.13) | <0.001 | 1.38 (1.03, 1.86) | 0.030 |
| Business | 1.82 (1.49, 2.22) | <0.001 | 1.94 (1.47, 2.54) | <0.001 | 1.28 (0.94, 1.74) | 0.112 |
| Self-employed | 1.29 (1.07, 1.56) | 0.008 | 1.57 (1.25, 1.96) | <0.001 | 1.02 (0.65, 1.59) | 0.944 |
| Housewife | 1.37 (1.17, 1.60) | <0.001 | 1.63 (1.32, 2.01) | <0.001 | 0.87 (0.67, 1.13) | 0.306 |
| Student | 1.16 (0.98, 1.37) | 0.900 | 1.23 (0.98, 1.55) | 0.075 | 0.81 (0.61, 1.06) | 0.122 |
| Others | 1.66 (1.17, 2.36) | 0.004 | 2.23 (1.42, 3.50) | 0.001 | 0.93 (0.53, 1.62) | 0.789 |
| **Income (BDT)** | | | | | | |
| <15,000 | 1 | | 1 | | 1 | |
| 15,000-19,000 | 0.96 (0.84, 1.11) | 0.624 | 0.98 (0.84, 1.14) | 0.791 | 0.76 (0.46, 1.24) | 0.266 |
| 20,000 or more | 1.11 (1.00, 1.21) | 0.400 | 0.86 (0.75, 0.98) | 0.026 | 0.93 (0.67, 1.28) | 0.645 |
| **Income reduced** | | | | | | |
| Yes | 1 | | 1 | | 1 | |
| No | 1.12 (1.02, 1.23) | 0.016 | 1.10 (0.94, 1.25) | 0.156 | 1.14 (0.99, 1.31) | 0.067 |

**Supplementary Table S2.** Bivariate logistic regression estimates of seropositive data matched with COVID-like symptoms by selected socio-demographic covariates.

| Factors | Seroprevalence Data Matched with COVID-like Symptoms | | | | | |
|---|---|---|---|---|---|---|
| | Combined | | Slum Area | | Non-Slum Area | |
| | OR (95% CI) | *p*-Value | OR (95% CI) | *p*-Value | OR (95% CI) | *p*-Value |
| **City** | | | | | | |
| Dhaka | 1 | | 1 | | 1 | |
| Chattogram | 0.97 (0.79, 1.19) | 0.788 | 1.07 (0.82, 1.41) | 0.610 | 0.70 (0.51, 0.95) | 0.024 |
| **Area** | | | | | | |
| Slum | 1 | | - | | - | |
| Non-slum area | 1.64 (1.39, 1.93) | <0.001 | - | | - | |
| **Age group (years)** | | | | | | |
| 10-17 | 1 | | 1 | | 1 | |
| 18-59 | 1.66 (1.35, 2.03) | <0.001 | 1.67 (1.30, 2.13) | 0.000 | 1.51 (1.06, 2.16) | 0.023 |
| 60 or more | 1.42 (0.99, 2.03) | 0.052 | 1.42 (0.90, 2.28) | 0.145 | 1.18 (0.67, 1.05) | 0.570 |
| **Sex** | | | | | | |
| Female | 1 | | 1 | | 1 | |
| Male | 1.04 (0.89, 1.22) | 0.632 | 0.98 (0.80, 1.19) | 0.833 | 1.26 (0.96, 1.65) | 0.100 |
| **Education (years of schooling)** | | | | | | |
| None | 1 | | 1 | | 1 | |
| 1-4 | 1.22 (0.94, 1.57) | 0.128 | 1.16 (0.89, 1.52) | 0.266 | 1.54 (0.65, 3.65) | 0.328 |
| 5-9 | 1.41 (1.14, 1.75) | 0.002 | 1.35 (1.07, 1.72) | 0.013 | 1.18 (0.59, 2.37) | 0.644 |
| 10 or more | 1.87 (1.49, 2.36) | <0.001 | 1.27 (0.86, 1.88) | 0.228 | 1.54 (0.78, 3.04) | 0.216 |
| **Occupation** | | | | | | |
| Not-working/unemployed | 1 | | 1 | | 1 | |
| Service | 2.20 (1.61, 2.99) | <0.001 | 2.33 (1.61, 3.37) | <0.001 | 1.31 (0.73, 2.37) | 0.365 |
| Business | 2.34 (1.64, 3.32) | <0.001 | 2.51 (1.62, 3.88) | <0.001 | 1.25 (0.66, 2.37) | 0.468 |
| Self-employed | 1.83 (1.31, 2.57) | <0.001 | 2.22 (1.52, 3.24) | <0.001 | 1.15 (0.47, 2.81) | 0.761 |
| Housewife | 1.70 (1.28, 2.26) | <0.001 | 1.75 (1.23, 2.48) | 0.002 | 0.93 (0.55, 1.60) | 0.808 |
| Student | 1.28 (0.96, 1.72) | 0.096 | 1.39 (0.97, 1.99) | 0.069 | 0.67 (0.39, 1.16) | 0.156 |
| Others | 1.14 (0.53, 2.46) | 0.735 | 2.20 (0.93, 5.20) | 0.074 | 0.12 (0.02, 1.00) | 0.051 |
| **Income (BDT)** | | | | | | |
| <15,000 | 1 | | 1 | | 1 | |
| 15,000-19,000 | 1.00 (0.77, 1.29) | 0.985 | 1.03 (0.79, 1.33) | 0.835 | 0.49 (0.16, 1.46) | 0.203 |
| 20,000 or more | 1.19 (1.00, 1.42) | 0.050 | 0.85 (0.68, 1.06) | 0.152 | 0.86 (0.42, 1.78) | 0.691 |
| **Income reduced** | | | | | | |
| Yes | 1 | | 1 | | 1 | |
| No | 0.99 (0.82, 1.18) | 0.908 | 0.95 (0.76, 1.19) | 0.682 | 1.00 (0.73, 1.38) | 0.986 |
